# Predictive Modeling and Experimental Validation of Magnetophoretic Delivery of Magnetic Nanocultures

**DOI:** 10.1021/acsmaterialslett.5c00753

**Published:** 2025-06-25

**Authors:** Rohit Chauhan, Huda Usman, Nitin Minocha, Mehdi Molaei, Tagbo H. R. Niepa, Meenesh R. Singh

**Affiliations:** † Department of Chemical Engineering, 14681 University of Illinois−Chicago, Chicago, Illinois 60607, United States; ‡ Department of Chemical Engineering, 6612 Carnegie Mellon University, Pittsburgh, Pennsylvania 15213, United States; § Department of Chemical and Biomolecular Engineering, 6572 University of Pennsylvania, Philadelphia, Pennsylvania 19104, United States; ∥ Department of Biomedical Engineering, Carnegie Mellon University, Pittsburgh, Pennsylvania 15213, United States

## Abstract

Magnetophoresis offers a powerful strategy for the targeted delivery of functional microcapsules. Here, we present a combined theoretical and experimental framework to predict the magnetophoretic transport of magnetic nanocultures–microcapsules embedded with magnetic nanoparticles and living cells. We derive a novel analytical expression for the terminal velocity of microcapsules under a spatially decaying magnetic field. The model incorporates magnetic and hydrodynamic forces in low Reynolds number regimes and predicts microcapsule velocity variations with nanoparticle size and field strength. Experimental validation using nanocultures containing nanoparticles 5, 10, and 20 nm in size confirms the model’s accuracy, with 10-nm particles showing optimal magnetophoretic response. The model also accounts for hindered motion at high microcapsule densities. This work provides a predictive tool for designing magnetically guided systems for microbial delivery, localization, and patterning, with applications in bioreactors, therapy, and engineered living materials.

## Graphical abstract

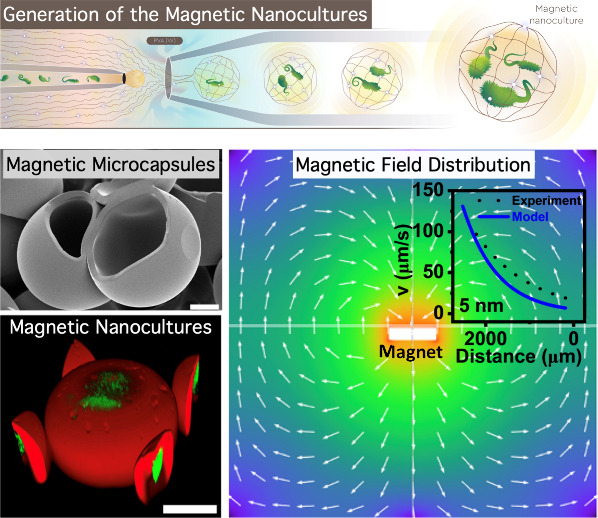

Nanocultures are nanoliter-scale microcapsules engineered to grow, store, and study microbial communities under controlled conditions.^1,2^ Recent advancements in culturomic technologies have enabled the integration of magnetic nanoparticles into these microstructures, resulting in the development of magnetic nanocultures.^2,3^ This innovation allows microbial systems to be remotely manipulated and spatially positioned using external magnetic fields. With magnetic functionality, precise spatial arrangement, real-time tracking, and localized stimulation, microcapsule behavior can be predicted. These capabilities are especially advantageous for in-situ cultivation within complex and heterogeneous environments, such as soil, gastrointestinal fluids, where conventional culturing methods often disrupt native microenvironments and fail to support the growth of many microorganisms. Through this platform, microbial consortia can be selectively deployed and retrieved, facilitating the study and isolation of previously unculturable or poorly understood microbes directly within their natural habitats.^3^

Here, we are evaluating magnetophoresis (the motion of magnetic objects under a field gradient) to remotely guide the magnetic nanocultures to specific locations.^3^ Using magnetophoresis , the nanocultures can be magnetically actuated, sorted, and isolated.^4–8^ These capabilities are significant for biomedical applications such as targeted drug delivery,^5–8^ localized cell therapy, and bioseparations, where magnetic forces direct therapeutic agents or cells to a desired site with high precision.^9–11^ Magnetophoresis-based targeting offers a noninvasive means to enhance the localization and retention of nanocultures in target environments, potentially improving metabolite exchange, community sorting, cell–cell communication across the shell of the nanocultures.^12–14^ Motivated by these advantages, the present study focuses on understanding and modeling the controlled transport of magnetic nanocultures under applied magnetic fields. The overall objective is to develop a predictive framework—combining mathematical modeling, simulation, and experiments—for the targeted delivery of these magnetically responsive microcapsules.

Previous studies on magnetophoretic transport have employed both numerical simulations^15–18^ and analytical modeling^19–21^ to characterize particle motion. Finite-element analysis (FEA) is often used to compute the magnetic field and resulting forces on particles, with the trajectories then obtained by integrating Newton’s equations of motion.^22^ However, such numerical approaches are computationally intensive and not well-suited for extensive parametric studies or design optimization.^23^ In contrast, analytical models provide closed-form solutions that offer precise insights into system behavior at every point in space, making them highly valuable for rapid design iterations.^21,23^ In a seminal work, Furlani^23^ developed an analytical model for magnetophoresis in a microfluidic device, deriving the governing equations of particle motion using explicit expressions for the magnetic and drag forces.^23^ This model demonstrated that a strong field gradient can robustly capture magnetic particles and even predicted an oscillatory particle trajectory as the particles traverse a spatially periodic field.^23^ While such studies significantly advanced the understanding of magnetophoretic dynamics, they did not provide a general closed-form expression for the *terminal magnetophoretic velocity* of a particle—i.e., its steady-state migration speed under a given magnetic field – as an explicit function of spatial position. In fact, a clear *spatially resolved* analytical formula for how a magnetic microcapsule’s velocity varies within a nonuniform field remains absent in the literature.

In this work, we address that knowledge gap by deriving a novel analytical expression for the terminal velocity of a magnetic microcapsule under an external field, explicitly capturing its dependence on spatial location. Starting from the balance of magnetic forces and viscous drag on a single microcapsule, we obtain a closed-form solution for the magnetophoretic velocity as a function of position in the field. This analytical solution is the first of its kind to reveals how the microcapsule’s speed increases or decreases with local field strength, providing important insight for device design and control. We further validate the model by performing experiments in which magnetic nanocultures are subjected to a controlled field, and their velocities are measured. The excellent agreement between theory and experiment confirms the accuracy of the derived formula. This work extends previous analytical frameworks by providing an explicit, position-dependent velocity prediction. It enables more-precise design calculations for magnetophoretic delivery systems.

To enable the development of theoretical models and simulations of microcapsule motion and transport under external magnetic fields, we first established a robust method for generating magnetically responsive microcapsules and nanocultures (Figure 1). Throughout this study, we use the term “microcapsules” to refer to structures with sterile water as the inner aqueous phase and “nanocultures” to refer to those with bacterial culture media in the core. As illustrated in Figure 1A, we synthesized a composite polymer matrix by combining vinyl-terminated PDMS with a hydride-functionalized PDMS cross-linker, a platinum catalyst, and iron oxide magnetic nanoparticles (MNPs). This matrix was the middle oil phase in a water-in-oil-in-water (W/O/W) emulsion system (Supporting Information).^3,14,24^

**Figure 1:**
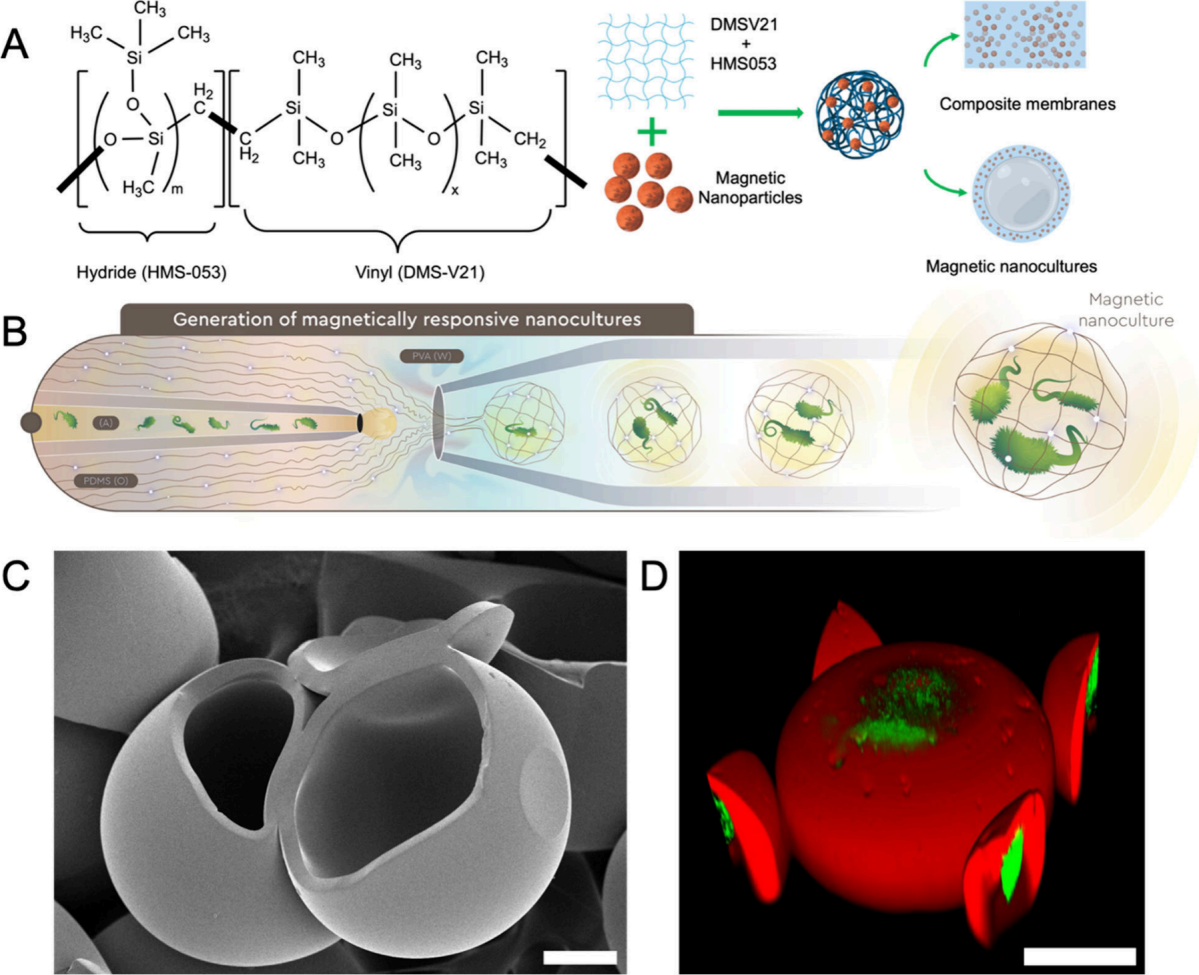
Development of magnetic microcapsules and nanocultures. (A) Schematic of the polymer cross-linking chemistry and the incorporation of magnetic nanoparticles into the PDMS matrix. (B) The three liquid phases (water for microcapsules and aqueous culture medium for nanocultures), actuated PDMS mixtures, and PVA solution) were introduced into the microfluidic device and directed into a three-phase interface for high-throughput generation of W/O/W emulsions. (C) Representative scanning electron microscopy image of magnetic microcapsules. Scale bar = 50 μm, (D) Confocal image of green fluorescent protein-tagged *P. aeruginosa* encapsulated in the magnetic nanocultures (MNCs). The polymeric shell of the MNCs was stained with lipophilic stain (Nile Red) to enhance image contrast. Scale bar = 50 μm.

Figure 1B presents a schematic of the three-phase coflow microfluidic process used to generate W/O/W double emulsions. The inner aqueous phase consisted of either sterile water (for microcapsules) or suspended in culture media (for nanocultures). The middle oil phase was the PDMS blend loaded with magnetic nanoparticles (MNPs), and the outer continuous phase was a 5 wt % aqueous solution of poly­(vinyl alcohol) (PVA). The resulting emulsions were pretreated by heating at 70 °C for 5 min and subsequently incubated at 37 °C for 24 h to form mechanically stable, semipermeable polymeric nanocultures.

Scanning electron microscopy (SEM) confirmed the successful formation of spherical, intact microcapsules with uniform membrane morphology (Figure 1C). The microcapsule size ranged from 180 μm to 200 μm, depending on the flow rates of the polymer and surfactant phases. Membrane integrity and bacterial viability were further validated using confocal fluorescence microscopy. Nanocultures were generated, collected in sterile culture media, and incubated at 37 °C for 24 h. Confocal imaging performed after incubation revealed confluent green fluorescent protein (GFP)-tagged cells encapsulated within the nanocultures (Figure 1D). The PDMS shell was stained with Nile Red to visualize the membrane boundary, clearly delineating the spatial separation between the encapsulated cells and the external environment.

This fabrication strategy provides the physical platform required to study magnetophoresis in complex systems. The known dimensions, permeability, and magnetic loading of the microcapsules serve as well-defined parameters for computational modeling of motion, fluid drag, and magnetic forces acting on individual microcapsules.

A key advantage of miniaturized culturing systems is their ability to support *in situ* and high-throughput microbial cultivation.^3^ To enable downstream retrieval following environmental deployment, we engineered nanoliter-scale culture systems (microcapsules) that remain free-floating in suspension (Figure 2). We hypothesized that these functionalized microcapsules could be magnetically actuated due to the superparamagnetic behavior of their PDMS-based membranes.^4^ Therefore, we next focused on measuring the magnetophoretic velocity to evaluate the microcapsules’ responsiveness to applied magnetic fields.

**Figure 2:**
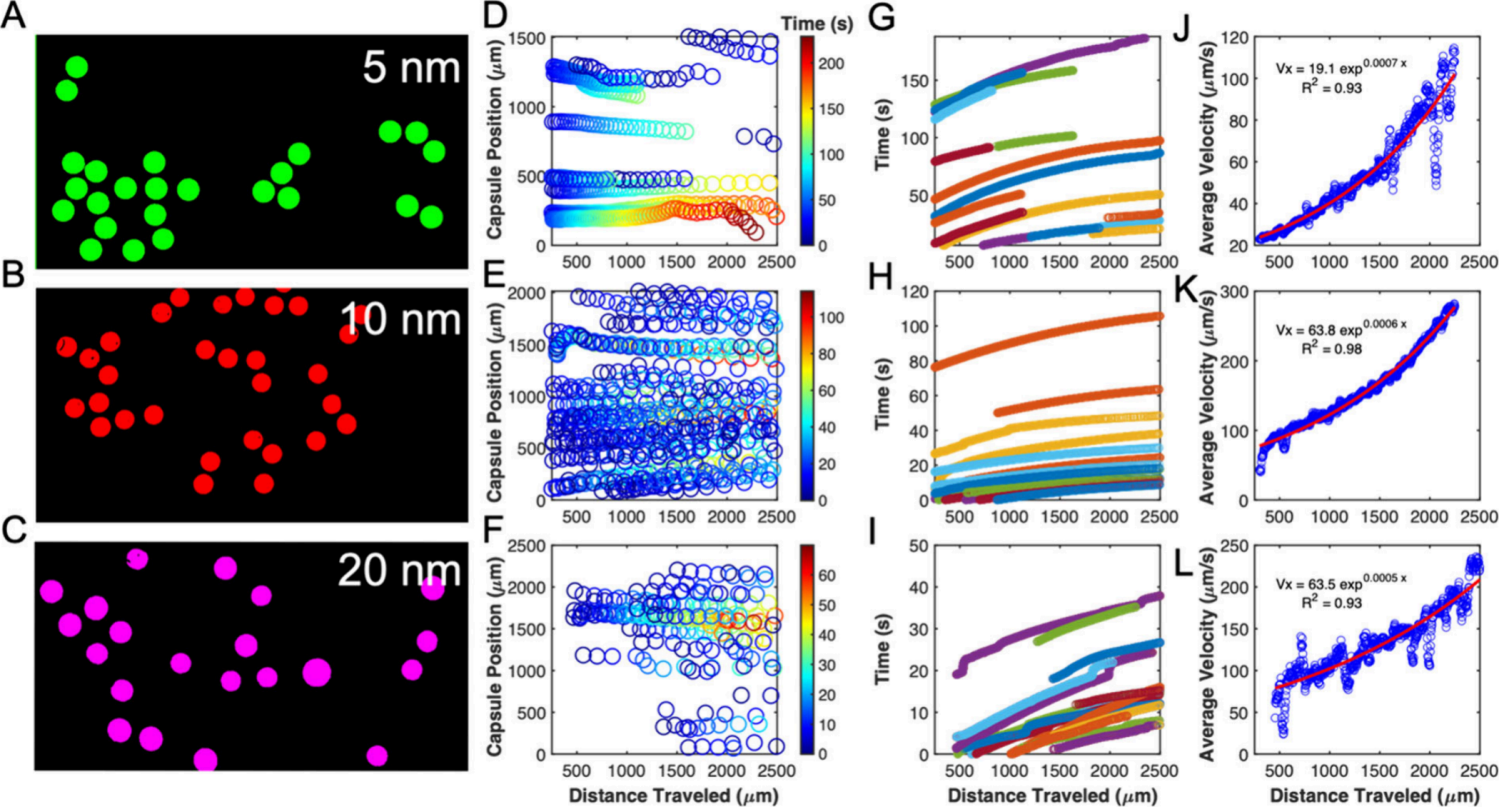
Magnetic actuation of microcapsules under a nonuniform magnetic. Panels (A–C) show microcapsules loaded with 500 ppm of 5-, 10-, or 20-nm iron oxide nanoparticles that were exposed to a nonuniform magnetic field. The first column shows raw ImageJ-processed images capturing the directional migration of microcapsules toward the magnet over time. Panels (D–F) display MATLAB-rendered trajectories colored by time, demonstrating microcapsule displacement as a function of distance from the magnet. Panels (G–I) present the corresponding velocity profiles extracted from tracked microcapsules, illustrating increasing velocity as the microcapsules approach the magnet. Panels (J–L) show curve-fitted average velocity versus distance data, revealing a nonlinear increase in magnetophoretic velocity consistent with the expected gradient-driven behavior. These results highlight the influence of nanoparticle size on microcapsule motion and were used to inform simulation parameters in subsequent modeling efforts. The number of individual MNCs tracked (*n*) was 12 for 5 nm, 15 for 10 nm, and 14 for 20 nm. [Figure reproduced from ref 3 under CC-BY 4.0 license. Copyright 2025.]

Superparamagnetic materials can be magnetized and demagnetized by applying or removing an external magnetic field, enabling precise control over their movement and colloidal stability.^25^ To evaluate magnetic responsiveness, we selected microcapsules containing the highest nanoparticle concentration (500 ppm), acknowledging that higher nanoparticle loading can compromise optical clarity. We further examined how nanoparticle size affects magnetically induced motion by fabricating microcapsules with 5-, 10-, and 20-nm magnetic nanoparticles, maintaining Milli-Q water as the internal and external medium (Figures 2A–C).

After overnight incubation at 37 °C to ensure complete cross-linking, the microcapsules were introduced into a SecureSeal hybridization chamber filled with Milli-Q water to eliminate osmotic gradients. For imaging, the chamber was mounted on a Nikon Eclipse TE300 inverted microscope, and a neodymium magnet (McMaster-Carr; ∼0.27 T surface field) was used to generate a magnetic field gradient. The movement was recorded using a Phantom VEO 710L high-speed camera at 24 fps.

Figures 2A–C display raw images showing the directional motion of microcapsules as they migrated toward the magnet (left to right). Figures 2D–I show MATLAB-rendered trajectories and displacement profiles over time, highlighting increasing velocity as microcapsules neared the magnetic source. Velocity data were extracted from individual microcapsule trajectories using a custom MATLAB tracking algorithm. In Figures 2J–L, the average velocities were found to be approximately 80 μm/s for 5 nm, 200 μm/s for 10 nm, and 130 μm/s for 20 nm nanoparticles. This capability is being further explored for applications involving dispersion in complex media such as wastewater sludge, marine water, and gastrointestinal fluids as part of our ongoing research.

Following the experimental evaluation of magnetophoretic velocity across different nanoparticle sizes, we next used simulations to investigate how variations in the static magnetic field influence microcapsule velocity and directional transport. The static magnetic field generated by a permanent magnet plays a pivotal role in determining the motion of magnetically functionalized microcapsules. The magnitude and spatial decay of the magnetic flux density directly influence the magnetic force experienced by the microcapsules and, consequently, their magnetophoretic velocity. Understanding the structure and gradient of the magnetic field is essential for accurately modeling and predicting nanoculture behavior in controlled environments. Figure 3A shows the magnetic flux density distribution around the magnet, indicating the strength and direction of the magnetic field as microcapsules move closer. This distribution is critical for understanding the forces acting on the nanoparticles embedded in the microcapsule shells. Figure 3B provides a detailed plot of the magnetic flux density along the narrow plate, demonstrating the exponential decay of the magnetic field strength as the distance from the magnet increases. This decay directly influences the magnetic force driving the microcapsules and is a key factor in our model’s velocity predictions.

**Figure 3:**
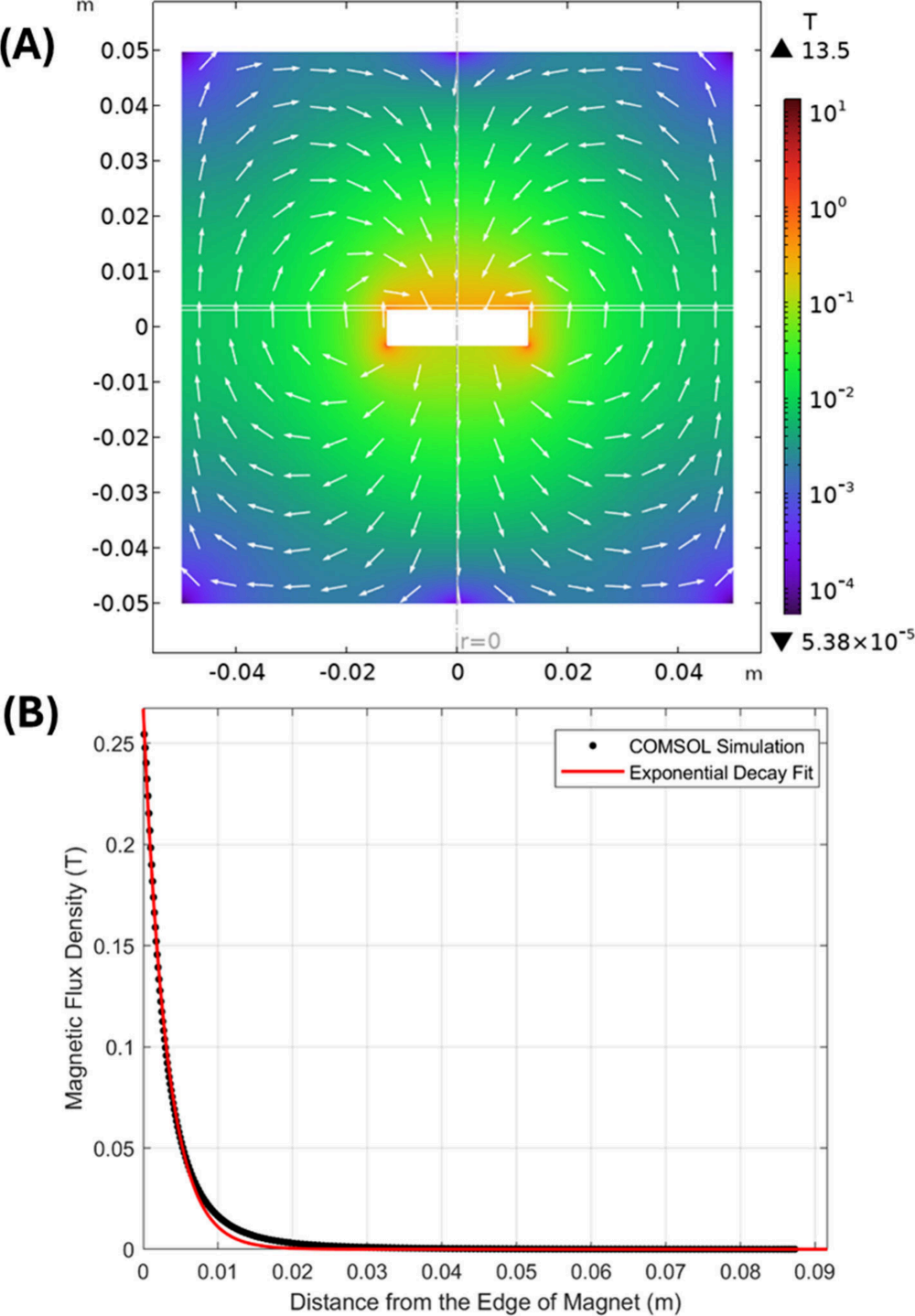
Influence of a static magnetic field. (A) The distribution of magnetic flux density around a magnet. The suspension of magnetic particles is placed on a narrow plate between two white horizontal lines on top of the magnet. (B) Magnetic flux density along the narrow plate and its fitting by an exponential decay function.

These findings highlight that the region of highest transport efficiency lies within a few millimeters of the magnet, where the gradient is strongest. The interplay between magnetic driving force and viscous drag reaches equilibrium at terminal velocity, which increases nonlinearly with field strength and is maximized near the magnet’s surface. The spatial distribution of the static magnetic field critically determines the efficiency of nanocultures motion. The exponential decay of magnetic flux with distance not only validates the theoretical assumptions used in our model but also emphasizes the importance of field geometry in designing magnetically guided delivery or sorting systems.

The magnetophoretic model for a single particle, presented in the Supporting Information, offers quantitative insight into the transport behavior of functionalized nanocultures under static magnetic fields. At steady state or negligible acceleration of the particle, the terminal velocity, *v*_*p*,*t*_, can be obtained from the balance of magnetic and drag forces in low Reynolds number (creeping flow) regimes, such as

1 $$v_{p,t}=\frac{V_{p}\chi_{p,\mathrm{eff}}}{3\pi \eta d_{p}\mu_{0}{\left(1+\chi_{p,\mathrm{eff}}\right)}^{2}}B_{a}\frac{dB_{a}}{dx}$$

The terminal velocity of the microcapsules is primarily governed by two magnet parameters, i.e., applied magnetic field strength (*B*_*a*_), and magnetic field gradient (captured by exponential decay function); and three other parameters related to particle, such as microcapsule volume (*V*_*p*_) and size (*d*_*p*_), and the effective magnetic susceptibility (χ_*p*,eff_) of the microcapsule, which accounts for the volume fraction of magnetizable particles in microcapsules, demagnetization of particles, and the susceptibility of the fluid medium, respectively. As microcapsules move through a spatially decaying magnetic field, the magnetic force increases significantly near the magnet, accelerating the particles. However, this motion is counteracted by fluidic drag, and a steady-state terminal velocity is eventually reached. Our model captures this behavior by integrating both the field gradient and fluid viscosity (η) into a single predictive framework.

Figure 4A illustrates the predicted terminal velocities for microcapsules exposed to increasing magnetic field strengths. The curve demonstrates a sharp, parabolic increase in velocity with field strength, consistent with the model’s prediction that magnetic force scales with the gradient of the square of the field. This finding confirms that even small increases in field intensity near the magnet surface can dramatically enhance the mobility of magnetic nanocultures. Figure 4B highlights the effect of varying effective susceptibility on microcapsule velocity. A clear linear relationship is observed, confirming that increasing the magnetic content of the nanocultures, for example by embedding a higher density or a more responsive type of magnetic nanoparticle, directly enhances their velocity under a constant field. This trend supports the utility of susceptibility as a tunable design parameter for magnetically guided systems.

**Figure 4:**
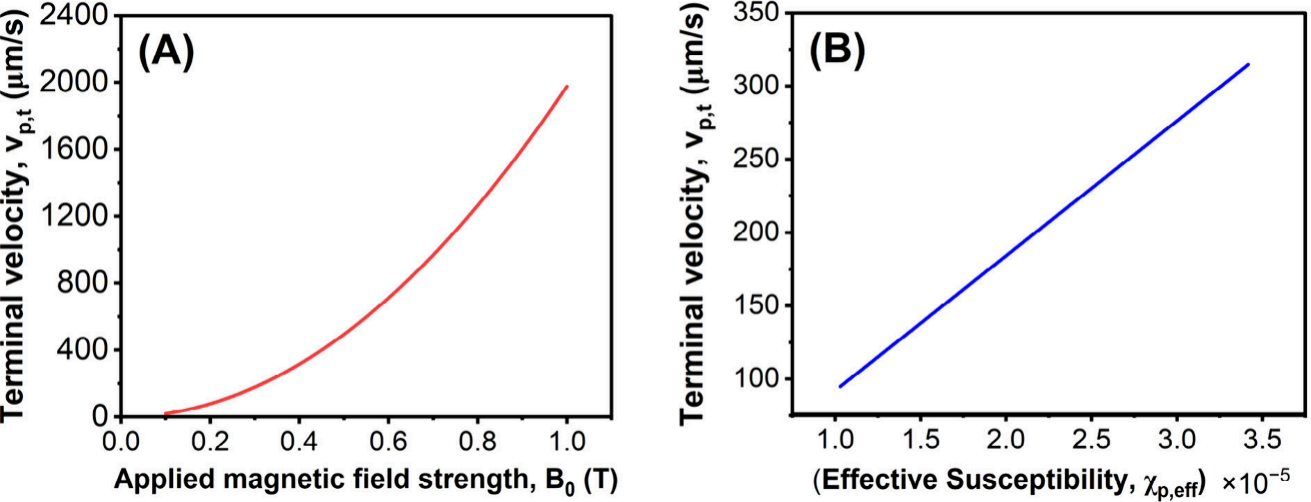
Effect of microcapsule/nanocultures’ physical properties on magnetophoretic velocity. Velocity distribution for (A) increasing applied magnetic field strength, and (B) increasing effective susceptibility.

These results provide predictive insight into the optimization of magnetically responsive delivery systems. For instance, designing nanocultures with tailored magnetic susceptibilities and deploying them in environments with engineered field gradients can significantly improve targeting efficiency and recovery rates.

The influence of nanoparticle size on nanoculture velocity was investigated to enable the prediction of their behavior in future applications. We incorporated the effects of nanoparticle size and concentration into the effective magnetic susceptibility. This susceptibility is used to estimate magnetophoretic velocities based on the size of nanoparticles embedded in microcapsules.

The magnetic force acting on paramagnetic microcapsules depends on several factors, including nanoparticle size, the gradient of the magnetic field, and the overall magnetic susceptibility of the microcapsule. As the field gradient increases exponentially near the magnet, the microcapsules accelerate; however, this movement is counteracted by the increasing fluidic drag. Our model balances these forces and predicts that the velocity of the microcapsules should increase steadily as they approach the magnet, with the highest velocities expected for intermediate nanoparticle sizes (e.g., 10 nm). Figure 5 compares the experimental and predicted velocity profiles for microcapsules containing 5-, 10-, and 20-nm nanoparticles, as well as 5-nm nanocultues containing bacteria. The dotted lines represent the experimental data, and the solid lines show the model’s predictions.

**Figure 5:**
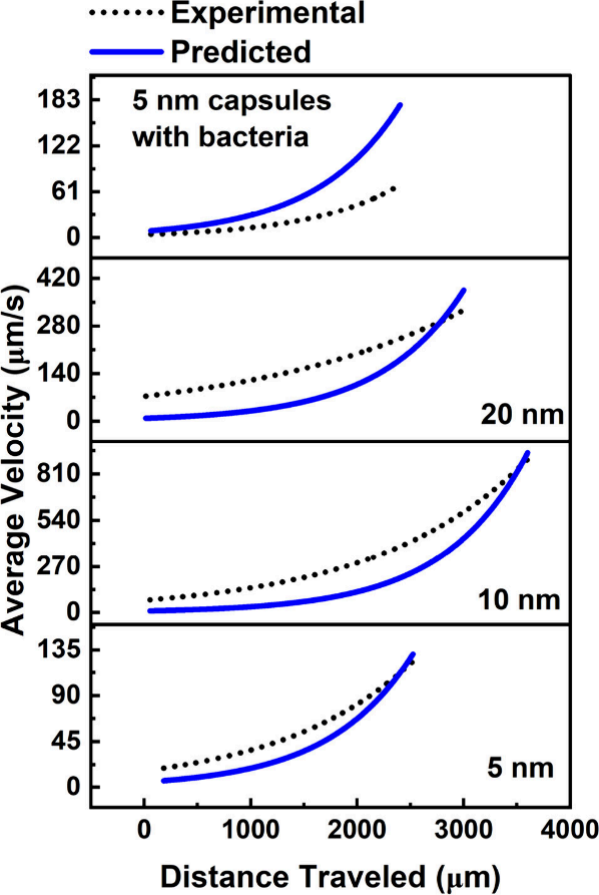
Validation of the theoretical model developed for velocity predictions. Experimental (dotted lines) and predicted (solid lines) velocity of the microcapsules with MNP of sizes 5-, 10-, and 20-nm nanoparticles, and 5-nm nanocultures with bacteria.

The theoretical model accurately predicts the increase in velocity (Figure 5) as microcapsules move closer to the magnet and highlights a nonmonotonous trend in velocity variation with increasing nanoparticle size. Microcapsules containing 10-nm nanoparticles exhibit the highest velocity due to their optimal magnetic susceptibility, whereas 5- and 20-nm nanoparticles result in lower velocities. This trend aligns with our expectations, as the 10-nm nanoparticles provide the best balance between magnetic responsiveness and drag.

The model’s predictions offer additional insights^4^ into how nanocultures embedded with different nanoparticle sizes behave under a magnetic field. This validation is particularly useful for future applications where nanocultures with varying sizes of nanoparticles may be used, enabling us to predict their velocities and optimize the system for targeted delivery or manipulation.

The comparison between experimental and predicted velocities demonstrates that our model can effectively capture the key dynamics of magnetophoretic motion, although minor deviations were observed at the beginning and end of the trajectories, possibly due to particle–particle interactions or drag wall. At these terminal points of the trajectories, the density of microcapsules is higher, causing significantly hindered motion. Such hindrance can be estimated using correlations discussed in the Supporting Information.

In conclusion, we developed a mathematical and experimental framework to predict and validate the targeted delivery of magnetic nanocultures using magnetophoresis. Our central contribution lies in deriving a novel analytical expression for the *terminal magnetophoretic velocity* of microcapsules under a spatially varying magnetic field. The velocity expression captures the dependence on the magnetic field and its gradients and provides spatially resolved predictions of particle velocity behavior. We validated this model experimentally using microcapsules embedded with magnetic nanoparticles of varying sizes and demonstrated excellent agreement with theoretical predictions. We found that microcapsules containing 10 nm particles achieved the highest velocities, reflecting an optimal tradeoff between magnetic responsiveness and hydrodynamic drag. This analysis also identified that particle–particle interactions at high local densities can result in hindered motion, which we estimated using semiempirical correlations. The derived terminal velocity expression serves as a valuable predictive tool for designing and optimizing magnetically guided delivery systems.

Looking forward, this framework can be extended in several impactful directions. First, incorporating time-varying or oscillating magnetic fields will enable the study of dynamic and programmable manipulation of magnetic nanocultures. Additionally, coupling the magnetophoretic model with nutrient transport and cellular activity could enable spatiotemporal control of living materials and engineered microbial consortia within host environments. Further experimental work involving 3D geometries and tissue-mimicking matrices will be crucial to transition from planar *in vitro* systems to *in vivo* or organ-on-chip platforms. Finally, the integration of this predictive framework with closed-loop control systems could facilitate precision delivery and programmable spatial patterning of living microcapsules in biomedical and biomanufacturing applications.

## Supplementary Material



## Notes

Details of experimental methods; calculation of the magnetophoresis of microcapsules and relevant equations (PDF)
