# Supplementary material for: Predictive Modeling and Experimental Validation of Magnetophoretic Delivery of Magnetic Nanocultures
Source: ACS Mater Lett. 2025 Jun 25;7(7):2679–85. doi: 10.1021/acsmaterialslett.5c00753 (PMC12239069; doi:10.1021/acsmaterialslett.5c00753)
Supplement: Supplementary file 1 [file tz5c00753_si_001.pdf]

Supporting Information

Predictive Modeling and Experimental Validation of Magnetophoretic Delivery of Magnetic Nanocultures

Rohit Chauhan[1#], Huda Usman[2#], Nitin Minocha[1], Mehdi Molaei[3], Tagbo H.R. Niepa[2,4*], and Meenesh R. Singh[1,#,*]

[1]Department of Chemical Engineering, University of Illinois Chicago, Chicago, IL, USA 60607

[2]Department of Chemical Engineering, Carnegie Mellon University, Pittsburgh, PA, USA 15213

[3]Department of Chemical and Biomolecular Engineering, University of Pennsylvania, Philadelphia, PA, USA 19104

[4]Department of Biomedical Engineering, Carnegie Mellon University, Pittsburgh, PA, USA 15213

# Authors contributed equally

*** Corresponding Authors:**

Prof. Meenesh R. Singh
Professor
Department of Chemical Engineering
929 W. Taylor St.
University of Illinois Chicago
Chicago, IL 60607
Tel: (312) 413-7673
Email: mrsingh@uic.edu

Prof. Tagbo Niepa
Associate Professor
Department of Chemical Engineering
5000 Forbes Avenue
Carnegie Mellon University
Pittsburgh, PA 15213
Email: tniepa@andrew.cmu.edu

Contents

Methods

Experimental Methods

Theoretical Methods

Table

Table S1. Properties of Microcapsules.

Table S2. Properties of Water and Magnet.

S1. Experimental Methods

S1.1. Generation of Magnetic Nanocultures for Microbial Encapsulation

Magnetic nanocultures (MNCs) were fabricated using a PDMS-based polymer matrix functionalized with iron oxide nanoparticles (5, 10, or 20 nm) to impart magnetic responsiveness as previously described.[1] The polymer was synthesized by mixing vinyl-terminated (DMS–V21) and hydride-functionalized (HMS–053) PDMS at a 0.6:1 molar ratio with 1.4 ± 0.4 ppm platinum catalyst using a previously established protocol.[2, 3] 500 ppm of 5 nm nanoparticles were used for optimal nanoculture stability and transparency. To form nanocultures, water-in-oil-in-water (W/O/W) emulsions were generated in a glass-capillary microfluidic device. The inner phase contained either sterile Milli-Q water (for background microcapsules) or a bacterial suspension (*E. coli Nissle* 1917 or GFP-tagged *P. aeruginosa* PAO1) in an ultrafiltered tryptone-yeast extract (UFTYE) medium. The middle phase consisted of the PDMS-MNP blend, and the outer phase was an aqueous 5 wt.% polyvinyl alcohol (PVA) solution to stabilize the double emulsions. Nanocultures were polymerized by heat-curing at 70 °C for 5 minutes and incubated at 37 °C for 24 hours. Encapsulation quality, stability, and microbial growth were monitored using bright-field and confocal microscopy (n=12 for 5 nm, n=15 10 nm, n=14 20nm).

S1.2. Magnetophoresis and Motion Tracking of Magnetic Nanocultures

To evaluate magnetophoretic behavior, 100–200 μL of polymerized magnetic nanocultures were added to hybridization collection chambers prefilled with sterile UFTYE media or Milli-Q water to match the internal composition and prevent osmotic disturbances. Chambers were mounted on an inverted microscope (Nikon Eclipse TE300), and a neodymium magnet (1.48 T, McMaster-Carr) was positioned externally to induce magnetic actuation. High-speed imaging (10,000 fps, Phantom VEO 710L) was used to capture nanoculture motion.

Image sequences were processed using custom MATLAB code. Magnetic nanocultures (MNCs) were identified using Otsu thresholding and tracked using the Crocker–Grier algorithm.[4, 5] Displacement data were converted into velocity profiles, and a median filter was applied to remove outliers. Velocity trends were analyzed as a function of spatial position within the magnetic gradient. Multiple independent experiments were performed for each nanoparticle size condition. The number of individual MNCs tracked was n = 12 for 5 nm, n = 15 for 10 nm, and n = 14 for 20 nm.

To calculate average velocities, particle trajectories were segmented frame-by-frame, and velocities were computed by dividing the x- and y-displacements by the time interval (24 fps), using a pixel-to-micron conversion. Capsules were then grouped into 10 +/- 2 μm-wide spatial bins along the x-axis. Mean V_x was calculated for each bin, excluding outliers that deviated more than one standard deviation from the median. The resulting average velocity and associated standard error were used to generate spatial velocity profiles across the magnetic field gradient. The properties of magnetic microcapsules are given in Table S1.

Table S1. Properties of Microcapsules.

**Size of capsules (m)**	0.000165	0.000167	0.000149	0.000243
**Size of MNP (nm)**	5	10	20	5 (with bacteria)
**Volume of shell (m^3)**	7.118 x 10^{-13}	8.205 x 10^{-13}	1.080 x 10^{-12}	1.603 x 10^{-12}
**Susceptibility of Capsule**	1.52 x 10^{-5}	2.44 x 10^{-5}	1.58 x 10^{-5}	1.52 x 10^{-5}
**Effective Susceptibility of Capsule**	1.52 x 10^{-5}	2.44 x 10^{-5}	1.58 x 10^{-5}	1.52 10^{-5}

S2. Theoretical Methods

S2.1. Modeling and Simulation of Magnetophoresis of a Single Particle

The particle transport under the influence of an external magnetic field in a magnetophoretic system depends on several forces, such as magnetic force, viscous drag, inertial effects, gravitational force, buoyancy, thermal kinetics, particle-fluid interaction, interparticle distance, magnetic-dipole interactions, and van der Waals forces. we consider a magnetic spherical particle of volume, V_p [m^3], moving towards a magnet of strength B_0 [T]. The study focuses on transporting magnetic micro/nanoparticles in creeping flow regimes. Therefore, the dominant forces acting on the particles are (i) the driving force due to the magnetic field gradient, $\vec{\mathbf{F}}_B$ [N], which acts on the particle toward the magnet, and (ii) the solution drag force, $\vec{\mathbf{F}}_D$ [N], acting in the direction opposite to the particle velocity. The balance of these two forces determines the particle's velocity.

i) Magnetic Force on a Single Microcapsule under Static Magnetic Field

The magnetic force acting on a magnetic particle is given by[5-7]

$$\vec{\mathbf{F}}_B = \mu_f V_p \left(\vec{\mathbf{M}}_p \cdot \vec{\nabla}\right)\vec{\mathbf{H}}_a \quad (1)$$

where $\mu_0 = 4\pi \times 10^{-7}$ [H/m] is the permeability of vacuum, χ_f and $\mu_f = \mu_0\left(1+\chi_f\right)$ are the susceptibility and permeability of the fluid medium, $\vec{\mathbf{H}}_a$ [A/m] is the applied magnetic field strength, and $\vec{\mathbf{M}}_p$ [A/m] is the magnetization (i.e., magnetic moment per unit volume) of a particle. In the case of magnetophoresis in aqueous solutions, the magnetic susceptibility of the medium can be considered negligible, such that $\left|\chi_f\right| << 1$ and $\mu_f \approx \mu_0$. Assuming a linear magnetization model,[8] the magnetization of a particle is directly proportional to the induced magnetic field strength.

$$\vec{\mathbf{M}}_p = \left(\chi_p - \chi_f\right)\vec{\mathbf{H}}_{in} \quad (2)$$

where $\chi_p = \mu_p / \mu_0 - 1$ and μ_p [H/m] are the susceptibility and permeability of a particle. For a composite particle, such as a microcapsule, susceptibility should account for the volume fraction of magnetically susceptible nanoparticles in the microcapsules.

$$\chi_p = c\,V_m\,\chi_n \tag{3}$$

where c is the molar concentration of nanoparticles in the microcapsule, V_m is the molar volume of the nanoparticles, which depends on their size, and χ_n is the volume susceptibility of the nanoparticles. In equation (2), $\vec{\mathbf{H}}_{in}$ is the induced magnetic field, which is the difference between the applied magnetic field $\left(\vec{\mathbf{H}}_a\right)$ and the self-demagnetization field generated by the particle $\vec{\mathbf{H}}_d = \vec{\mathbf{M}}_p/3$. The induced magnetic field strength can be related to the applied field strength as follows

$$\vec{\mathbf{H}}_{in} = \frac{3}{\left(\chi_p - \chi_f\right)+3}\vec{\mathbf{H}}_a \tag{4}$$

Substituting (4) in (2) gives

$$\vec{\mathbf{M}}_p = \frac{3\left(\chi_p - \chi_f\right)}{\left(\chi_p - \chi_f\right)+3}\vec{\mathbf{H}}_a \tag{5}$$

The proportionality constant in this equation can be considered as the effective susceptibility of a capsule, which accounts for demagnetization and the susceptibility of the fluid medium.

$$\chi_{p,eff} = \frac{3\left(\chi_p - \chi_f\right)}{\left(\chi_p - \chi_f\right)+3} \tag{6}$$

In the case of ferrimagnetic particles suspended in an aqueous solution, $\chi_p - \chi_f \approx \chi_p$. Therefore, eq (6) can be simplified as

$$\chi_{p,eff} = \frac{3\chi_p}{\chi_p + 3} \tag{7}$$

Substituting (5) in the magnetic force expression (1) gives,

$$\vec{\mathbf{F}}_B = \mu_0\,V_p\,\chi_{p,eff}\left(\vec{\mathbf{H}}_a \cdot \vec{\nabla}\right)\vec{\mathbf{H}}_a \tag{8}$$

The magnetic force expression can also be written in terms of applied magnetic flux density, $\vec{\mathbf{B}}_a$ [T], using relation

$$\vec{\mathbf{B}}_a = \mu_0\left(\vec{\mathbf{H}}_a + \vec{\mathbf{M}}_p\right) = \mu_0\left(1+\chi_{p,eff}\right)\vec{\mathbf{H}}_a = \mu_{p,eff}\,\vec{\mathbf{H}}_a \tag{9}$$

in equation (8), such that

$$\vec{\mathbf{F}}_B = \frac{V_p\,\chi_{p,eff}}{\mu_0\left(1+\chi_{p,eff}\right)^2}\left(\vec{\mathbf{B}}_a \cdot \vec{\nabla}\right)\vec{\mathbf{B}}_a \tag{10}$$

In one dimension,

$$F_B = \frac{V_p \chi_{p,eff}}{\mu_0 \left(1 + \chi_{p,eff}\right)^2} B_a \frac{dB_a}{dx} \tag{11}$$

Drag Force

The fluidic drag force acting on a spherical particle moving in a stagnant fluid can be obtained from Stokes' law, such that

$$F_D = 3\pi \eta d_p v_p \tag{12}$$

where η is the viscosity of the fluid, d_p is the diameter of the particle, and v_p is the velocity of the particle.

Force Balance

A one-dimensional force balance on a particle can be written as follows

$$\rho_p V_p \frac{dv_p}{dt} = F_B - F_D \tag{13}$$

where ρ_p is the density of the particle. Here, we have neglected gravitational forces since the particles under consideration are microcapsules that have densities similar to the fluid. Substituting magnetic and drag forces in the force balance equation yields,

$$\rho_p V_p \frac{dv_p}{dt} = \frac{V_p \chi_{p,eff}}{\mu_0 \left(1 + \chi_{p,eff}\right)^2} B_a \frac{dB_a}{dx} - 3\pi \eta d_p v_p \tag{14}$$

The magnetic field B_a and its gradient are both independent of time. The magnetic field can be obtained from solving the conservation equation of magnetostatics.

$$\vec{\nabla} \cdot \vec{\mathbf{B}} = 0 \tag{15}$$

Magnetophoretic Velocity

In a one-dimensional system, the decay in the magnetic field can be expressed as

$$B = B_0 e^{-\alpha x} \tag{16}$$

where α [1/m] is the decay factor and B_0 [T] is the strength of the magnet. The value of α estimated from the exact solution of (15) is close to 318.067 [1/m] in the direction parallel to the magnet, and the applied magnetic field strength at the edge is B_0 = 0.266 T.

At steady state or negligible acceleration of the particle, the terminal velocity, $v_{p,t}$, can be obtained from the balance of magnetic and drag forces, such as

$$v_{p,t} = \frac{V_p \chi_{p,eff}}{3\pi \eta d_p \mu_0 \left(1 + \chi_{p,eff}\right)^2} B_a \frac{dB_a}{dx} \tag{17}$$

Unlike sedimentation, the terminal velocity increases exponentially as the particle approaches a magnet. However, this is an approximate equation. The acceleration of a particle due to a steady increase in velocity must be accounted for in an accurate estimation. It requires solving equation (14). First, we will write the governing equation in dimensionless quantities as follows

$$\frac{d\upsilon_p}{d\tau} = -\lambda_0 l_p e^{-2\alpha x} - \frac{18}{\mathrm{Re}_p}\upsilon_p \tag{18}$$

where $\lambda_0 = \frac{E_{magnetic}}{E_{kinetic}} = \frac{\chi_{p,eff} B_0^2 / 2\mu_0}{\rho_p v_{p,t}^2 / 2}$ is the ratio of magnetic energy to kinetic energy, $l_p = \alpha\, d_p$ is the dimensionless particle diameter, $\mathrm{Re}_p = \frac{d_p\, v_{p,t}\, \rho_p}{\eta}$ is the particle Reynolds number, $\upsilon_p = \frac{v_p}{v_{p,t}}$ is the dimensionless velocity, and $\tau = \frac{t\, v_{p,t}}{d_p}$ is the dimensionless time. In writing the dimensionless equation, we have considered $\left|\chi_{p,eff}\right| \ll 1$. Since the magnetic field and its gradient are independent of time, we can integrate the equation (18) to obtain a time-dependent velocity expression.

$$\upsilon_p = -\frac{\mathrm{Re}_p\, \lambda_0 l_p e^{-2\alpha x}}{18}\left(1 - e^{\frac{-18\tau}{\mathrm{Re}_p}}\right) \tag{19}$$

The particle Reynolds number in magnetophoresis is much less than 0.1, and the flow regime is close to creeping flow. In such scenarios, the exponent in the equation (19) is $18/\mathrm{Re}_p \gg 1$ that allow quick attainment of the steady state. Neglecting the time-dependent term, the expression of particle velocity can be written as

$$v_p = -\frac{\mathrm{Re}_p\, \lambda_0 l_p e^{-2\alpha x}}{18} v_{p,t} \tag{20}$$

The negative sign represents the direction of velocity, which is opposite to the direction of the magnetic field gradient.

The model parameters are given in Table S2.

Table S2. Properties of Water and Magnet.

Property	Value
**Viscosity**	0.00089 (Pa.s)
**Susceptibility**	-9.04×10^{-6}
**Permeability of Vacuum**	1.26×10^{-6} (H/m)
**B_0**	0.266 (T)
**Decay factor**	318.07 (m^{-1})

Hindered Magnetophoretic Velocity

The free motion behavior of a single spherical particle in a stagnant Newtonian fluid is given by equation (14). As the number of particles in the suspension increases, interaction between the particles becomes significant. Consequently, the terminal velocity of a particle in a liquid suspension is affected by the presence of nearby particles, and the drag force created by the phoretic particles will affect the movement of nearby particles, and magnetophoresis will be hindered.

Richardson and Zaki (1954) equation is the most widely used semi-empirical correlation for high volume fractions of particles ($0.05 < \phi < 0.50$) and for slightly polydisperse suspensions:

$$v_{p,h} = \left(1-\phi\right)^n v_{p,t} \tag{21}$$

where ϕ is the volume fraction of particles in the suspension, $v_{p,h}$ is the hindered terminal velocity, and n is the exponent determined by the particle Reynold's number.

Magnetophoretic Motion in a Flowing Medium

The magnetophoretic motion of particles can be influenced by the flowing medium and vice versa. To model the effect of particle motion on the fluid flow, we include the magnetic force term (Eq. (10)) in the Navier-Stokes equation

$$\frac{\partial \vec{\mathbf{v}}}{\partial t} + (\vec{\mathbf{v}}.\vec{\nabla})\vec{\mathbf{v}} = v\nabla\vec{\mathbf{v}} - \frac{\vec{\nabla}P}{\rho} + \vec{g} + \frac{\vec{\mathbf{F}}_m}{\rho} \tag{22}$$

where $\vec{\mathbf{v}}$ is the fluid velocity vector, v is the kinematic viscosity, P is the hydraulic pressure, $\vec{g}$ is the gravitational force, and ρ is the fluid density. The dependence of the solution flow on the concentration gradient of the particles/capsules requires coupling the solution flow transport equations with the particle transport equation. The continuity equation for particle transport is given by

$$(\vec{\nabla}.\vec{\mathbf{N}}) = 0 \tag{23}$$

More details regarding the modeling and simulation of magnetic particles in a flowing medium can be found in Ayansiji et al. [9]

References

(1) Usman, H.; Molaei, M.; House, S.; Haase, M. F.; Dennis, C. L.; Niepa, T. H. Magnetically Responsive Nanocultures for Direct Microbial Assessment in Soil Environments. *bioRxiv* **2025**, 2025.2005. 2017.654660.

(2) Manimaran, N. H.; Usman, H.; Kamga, K. L.; Davidson, S.-L.; Beckman, E.; Niepa, T. H. Developing a functional poly (dimethylsiloxane)-based microbial nanoculture system using dimethylallylamine. *ACS Applied Materials & Interfaces* **2020**, *12* (45), 50581-50591.

(3) Usman, H.; Davidson, S.-L.; Manimaran, N. H.; Nguyen, J. T.; Bah, A.; Seth, R.; Beckman, E.; Niepa, T. H. Design of a well-defined poly (dimethylsiloxane)-based microbial nanoculture system. *Materials Today Communications* **2021**, *27*, 102185.

(4) Crocker, J. C.; Grier, D. G. Methods of digital video microscopy for colloidal studies. *Journal of colloid and interface science* **1996**, *179* (1), 298-310.

(5) Otsu, N. A threshold selection method from gray-level histograms. *Automatica* **1975**, *11* (285-296), 23-27.

(6) Jones, T. B. Electromechanics of particles. *Cambridge University Press* **1995**. DOI: 10.1017/CBO9780511574498.

(7) Ayansiji, A. O.; Dighe, A. V.; Linninger, A. A.; Singh, M. R. Constitutive relationship and governing physical properties for magnetophoresis. *Proceedings of the National Academy of Sciences* **2020**, *117* (48), 30208-30214.

(8) Dielectrophoresis and magnetophoresis. In *Electromechanics of Particles*, Jones, T. B. Ed.; Cambridge University Press, 1995; pp 34-82.

(9) Ayansiji, A. O.; Dighe, A. V.; Linninger, A. A.; Singh, M. R. Constitutive relationship and governing physical properties for magnetophoresis. *Proceedings of the National Academy of Sciences* **2020**, 202018568. DOI: 10.1073/pnas.2018568117.
